# Supplementary material for: Microfluidic Preparation of 89Zr-Radiolabelled Proteins by Flow Photochemistry
Source: Molecules. 2021 Feb 2;26(3):764. doi: 10.3390/molecules26030764 (PMC7867232; doi:10.3390/molecules26030764)

Electronic Supporting Information (ESI)

Microfluidic preparation of ^{89}Zr-radiolabeled proteins by flow photochemistry

Daniel F. Earley,[1] Amaury Guillou,[1] Dion van der Born,[2], Alex J. Poot,[3] and Jason P. Holland[1]*

1. Department of Chemistry, University of Zurich, Winterthurerstrasse 190, CH-8057, Zurich, Switzerland
2. FutureChemistry Agro Business Park 10, 6708 PW, Wageningen, The Netherlands
3. Department of Radiology and Nuclear Medicine, UMC Utrecht, Heidelberglaan 100, 3584 CX Utrecht, The Netherlands

* **Corresponding Author:**
Prof. Dr Jason P. Holland
Tel: +41.44.63.53.990
E-mail: jason.holland@chem.uzh.ch
Website: www.hollandlab.org

First Author:
Daniel F. Earley
Email: daniel.earley@chem.uzh.ch

Table of Contents

Figure S1. ^{1}H (400 MHz, $CDCl_3$, 298 K) NMR spectrum of compound **2** 3
Figure S2. $^{13}C\{^{1}H\}$ (101 MHz, $CDCl_3$, 298 K) NMR spectrum of compound **2** 3
Figure S3. HRMS (negative mode) spectrum of compound **2** 3
Figure S4. ^{1}H (400 MHz, $CDCl_3$, 298 K) NMR spectrum of compound **4** 4
Figure S5. $^{13}C\{^{1}H\}$ (101 MHz, $CDCl_3$, 298 K) NMR spectrum of compound **4** 4
Figure S6. HRMS spectrum of compound **4** 4
Figure S7. ^{1}H (400 MHz, $CDCl_3$, 298 K) NMR spectrum of compound **5** 5
Figure S8. $^{13}C\{^{1}H\}$ (101 MHz, $CDCl_3$, 298 K) NMR spectrum of compound **5** 5
Figure S9. HRMS spectrum of compound **5** 5
Figure S10. ^{1}H (400 MHz, MeOD, 298 K) NMR spectrum of compound **6** 6
Figure S11. $^{13}C\{^{1}H\}$ (101 MHz, MeOD, 298 K) NMR spectrum of compound **6** 6
Figure S12. HRMS (negative mode) spectrum of compound **6** 6
Figure S13. ^{1}H (500 MHz, DMSO-d^6, 298 K) NMR spectrum of compound **1** 7
Figure S14. $^{13}C\{^{1}H\}$ (125 MHz, DMSO-d^6, 298 K) NMR spectrum of compound **1** 7
Figure S15. 2D-COSY (DMSO-d^6) NMR spectrum of compound **1** 8
Figure S16. 2D-hetereonuclear HSQC (DMSO-d^6) NMR spectrum of compound **1** 8
Figure S17. HRMS spectrum of compound **1** 9
Supporting Results 9
^{89}Zr-Radiolabelling of DFO-PEG$_3$-Et-ArN$_3$ (**1**) 9
Figure S18. Characterisation data for the radiochemical synthesis of [^{89}Zr]ZrDFO-PEG$_3$-Et-ArN$_3$. A) Radio-iTLC chromatograms of 'free' ^{89}Zr complexed as [^{89}Zr]Zr-DTPA (blue) and [^{89}Zr]ZrDFO-PEG$_3$-Et-ArN$_3$ (black); B) HPLC chromatograms of the ligand (compound **1**; blue), [^{nat}Zr]ZrDFO-PEG$_3$-Et-ArN$_3$ (green) and [^{89}Zr]ZrDFO-PEG$_3$-Et-ArN$_3$ (black). 9
References .... **Error! Bookmark not defined.**

Figure S1. *^{1}H (400 MHz, $CDCl_3$, 298 K) NMR spectrum of compound **2***

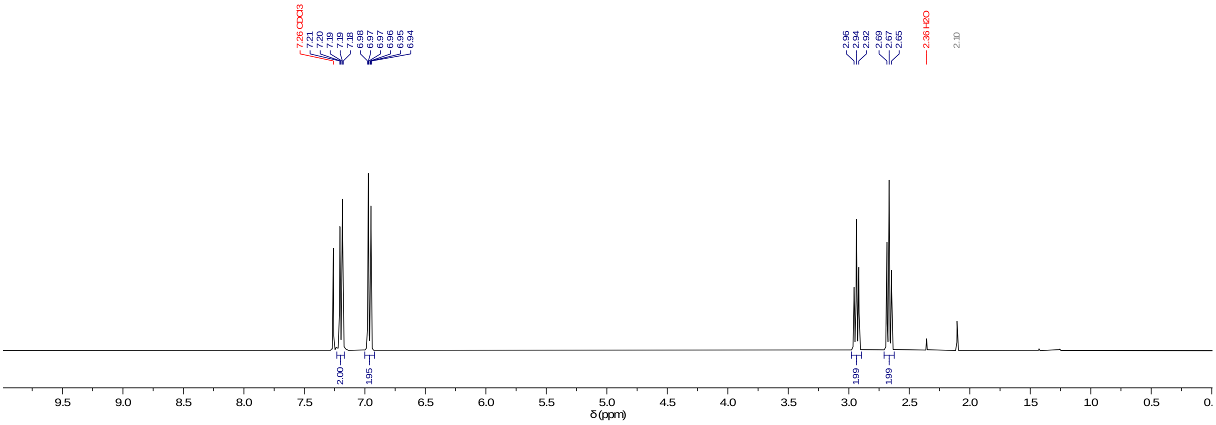

Figure S2. *$^{13}C\{^{1}H\}$ (101 MHz, $CDCl_3$, 298 K) NMR spectrum of compound **2***

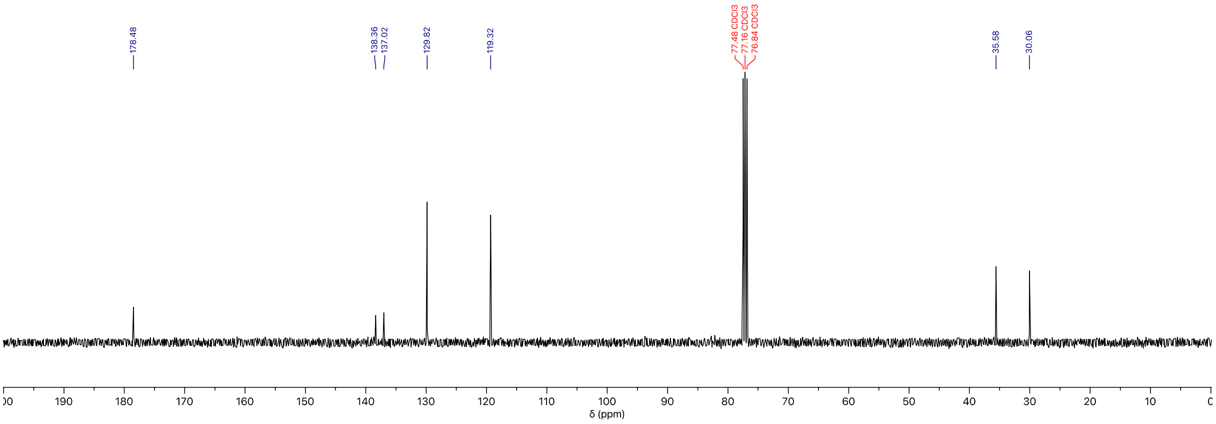

Figure S3. *HRMS (negative mode) spectrum of compound **2***

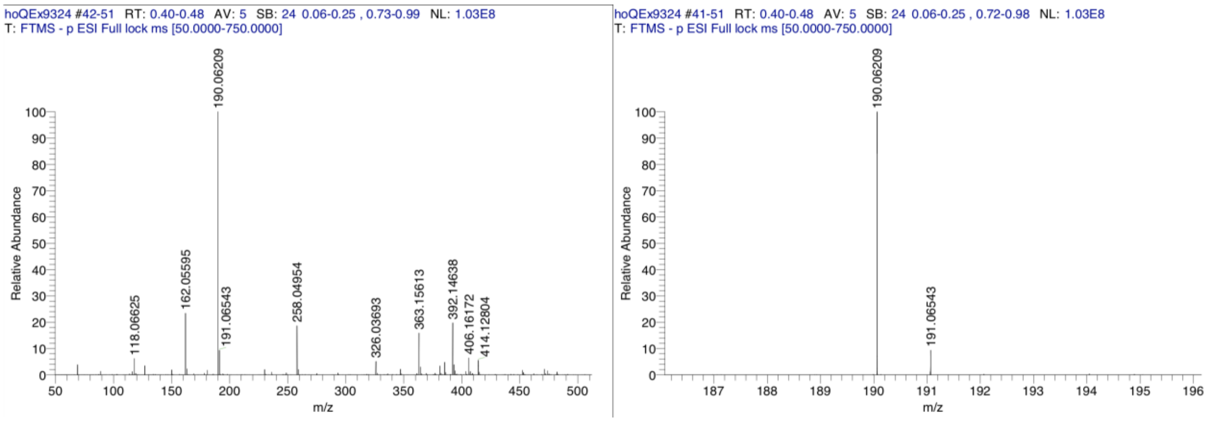

Figure S4. *^{1}H (400 MHz, $CDCl_3$, 298 K) NMR spectrum of compound* ***4***

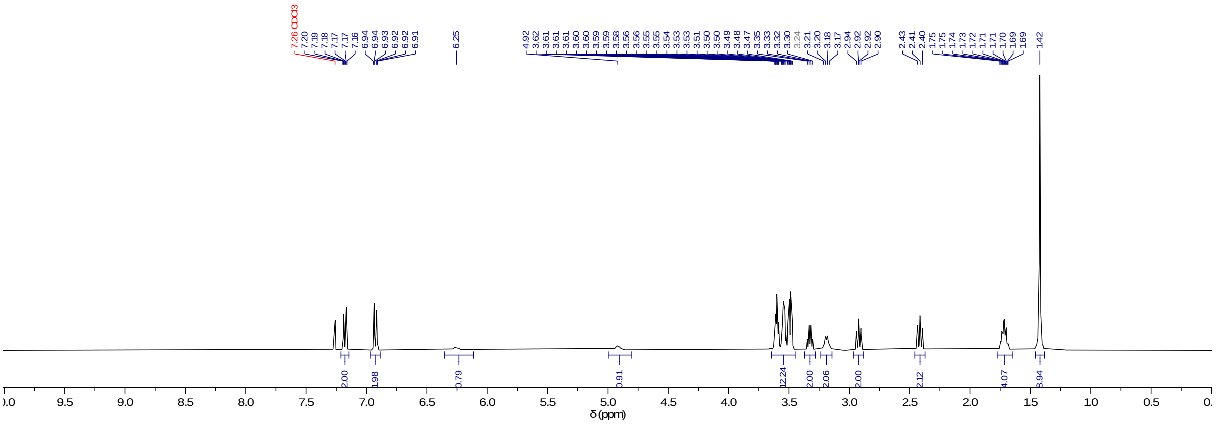

Figure S5. *^{13}C{^{1}H} (101 MHz, $CDCl_3$, 298 K) NMR spectrum of compound* ***4***

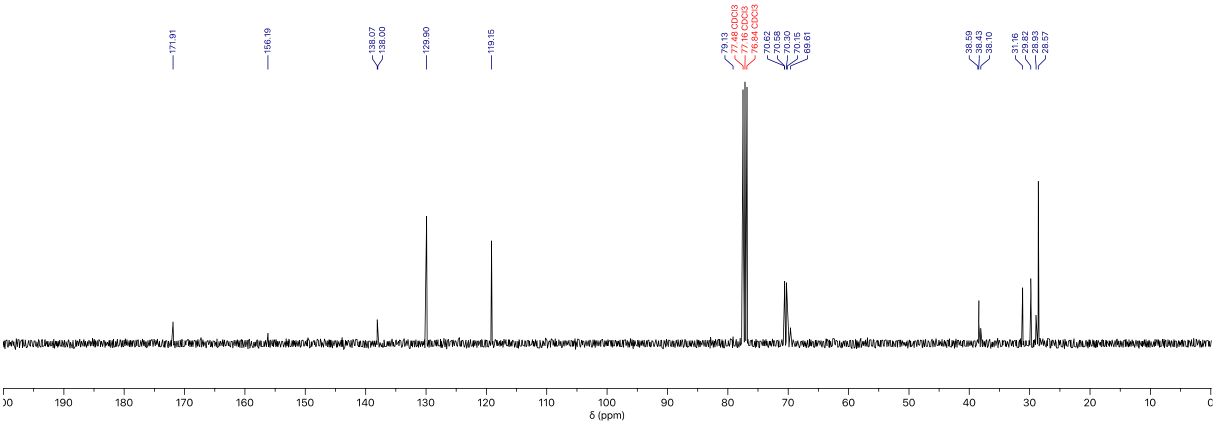

Figure S6. *HRMS spectrum of compound* ***4***

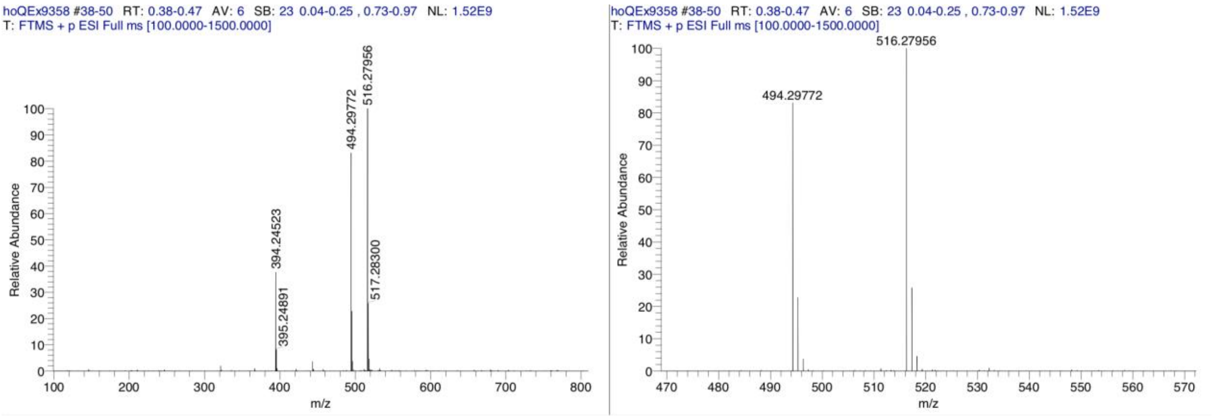

Figure S7. *^{1}H (400 MHz, $CDCl_3$, 298 K) NMR spectrum of compound* ***5***

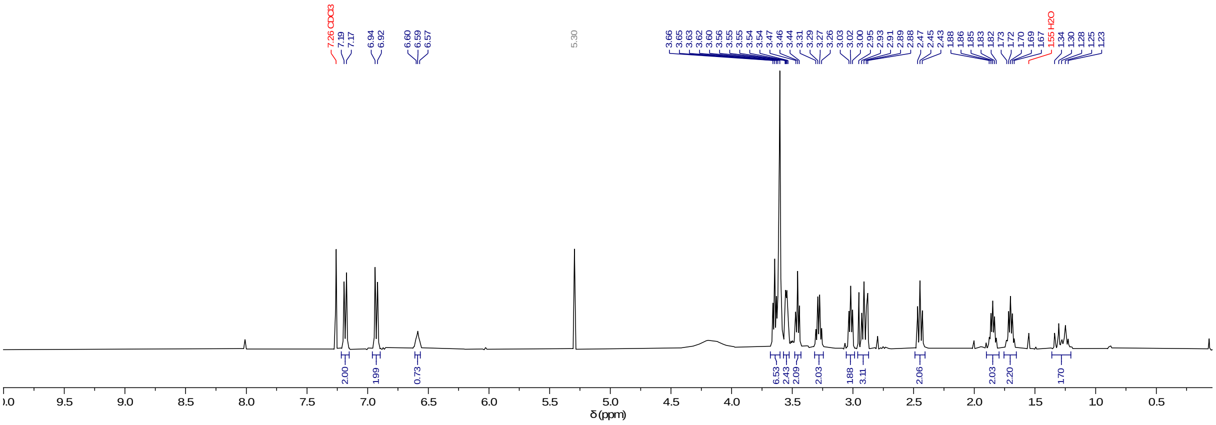

Figure S8. *^{13}C{^{1}H} (101 MHz, $CDCl_3$, 298 K) NMR spectrum of compound* ***5***

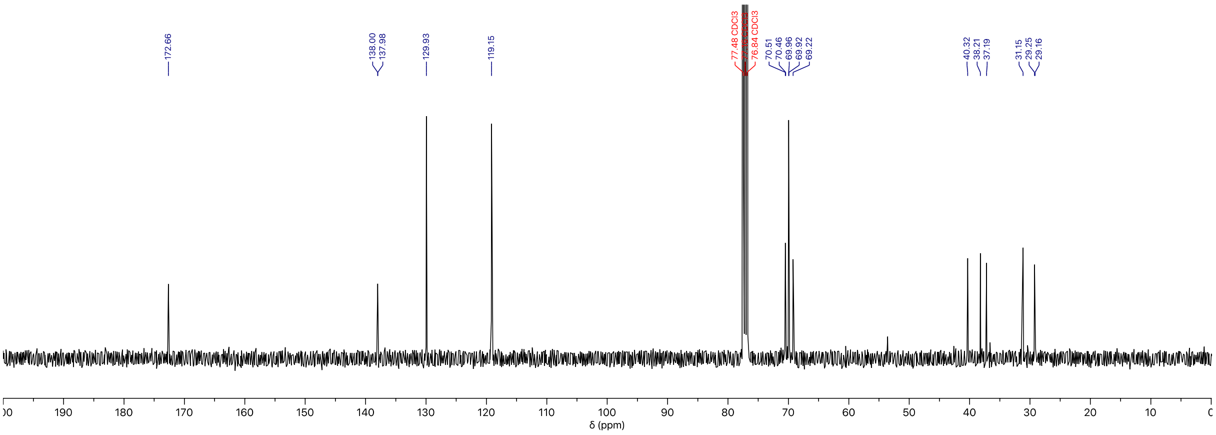

Figure S9. *HRMS spectrum of compound* ***5***

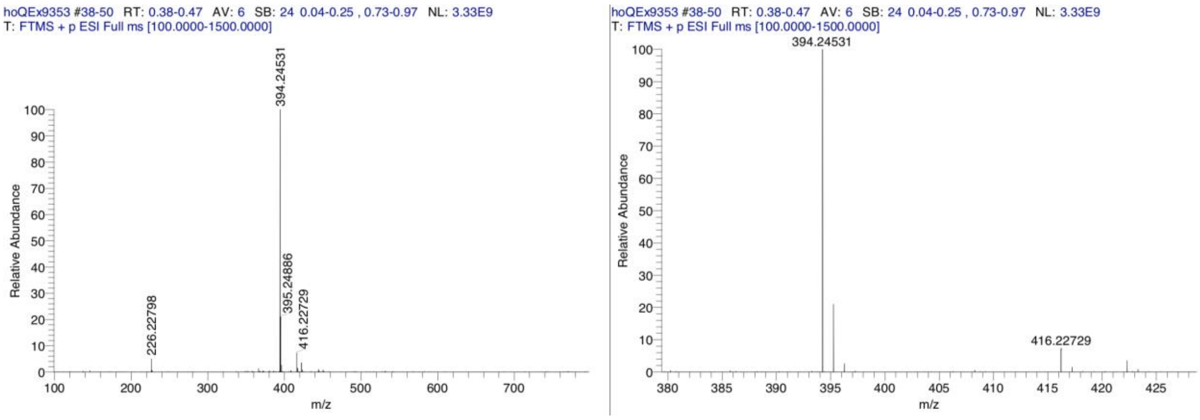

Figure S10. ^{1}H *(400 MHz, MeOD, 298 K) NMR spectrum of compound* ***6***

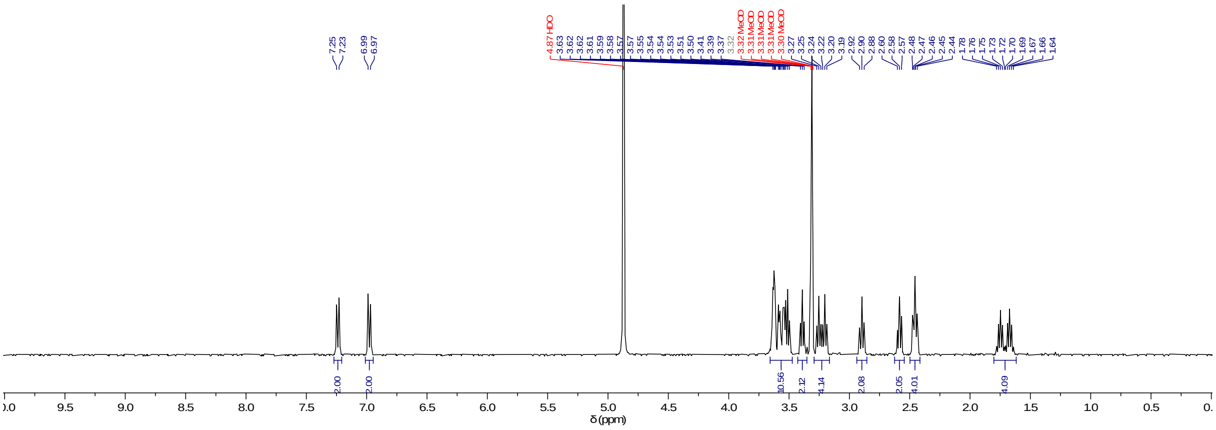

Figure S11. $^{13}C\{^{1}H\}$ *(101 MHz, MeOD, 298 K) NMR spectrum of compound* ***6***

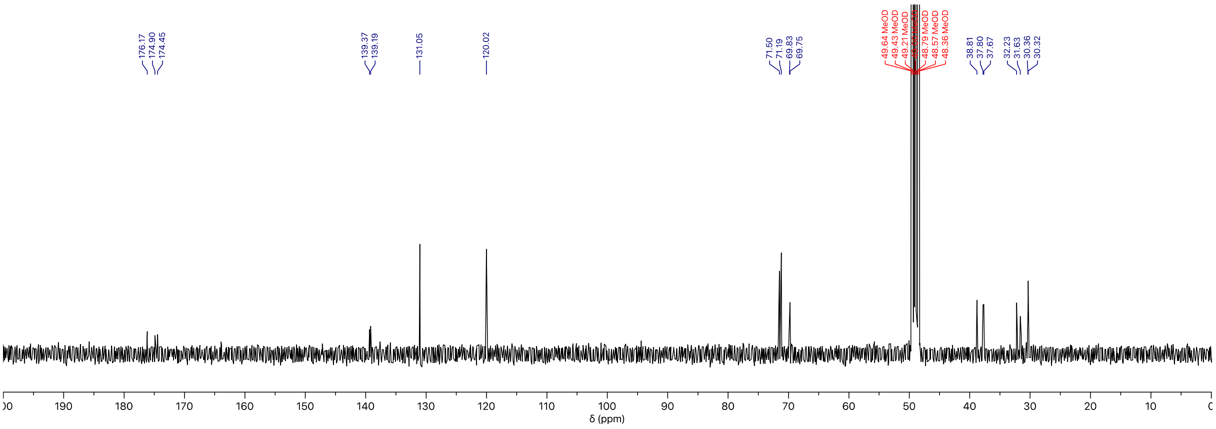

Figure S12. *HRMS (negative mode) spectrum of compound* ***6***

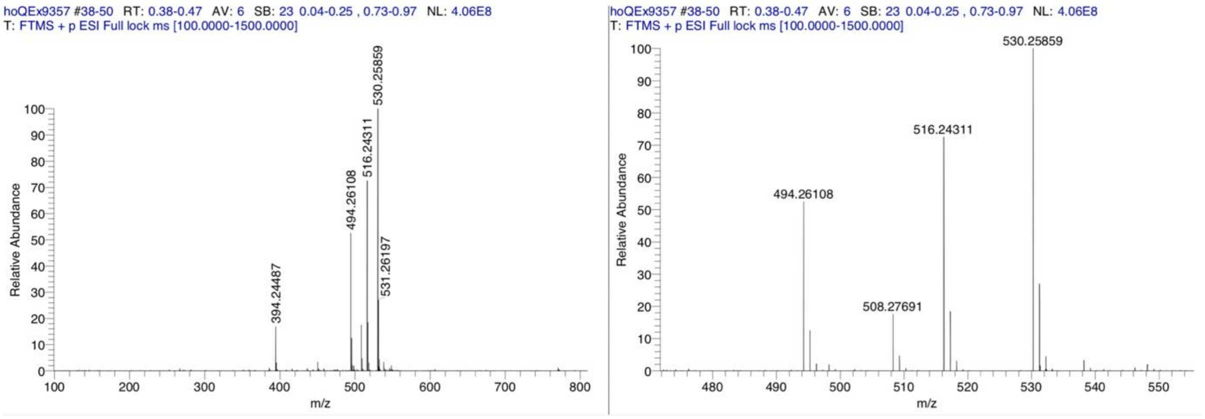

Figure S13. ^{1}H (500 MHz, dmso-d^{6}, 298 K) NMR spectrum of compound **1**

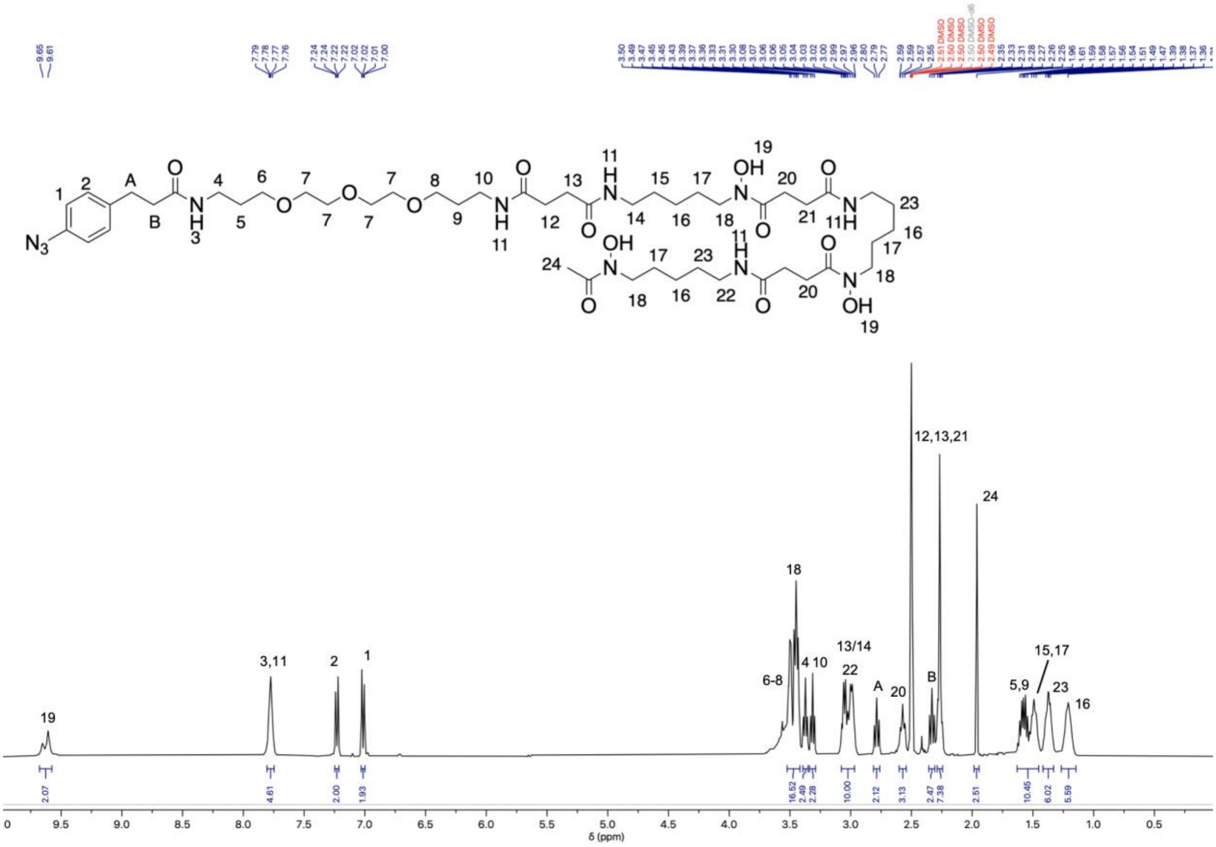

Figure S14. $^{13}C\{^{1}H\}$ (125 MHz, dmso-d^{6}, 298 K) NMR spectrum of compound **1**

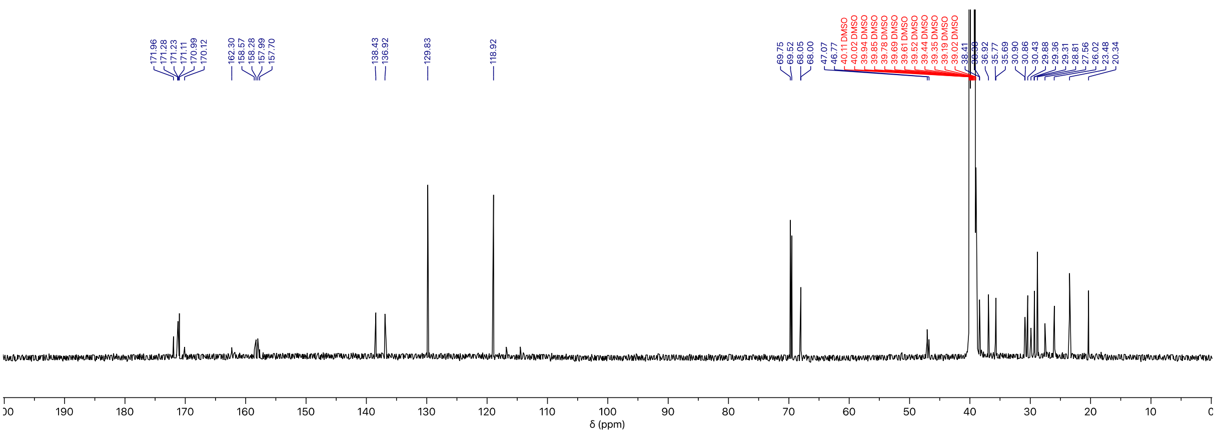

Figure S15. *2D-COSY (dmso-d^6) NMR spectrum of compound* ***1***

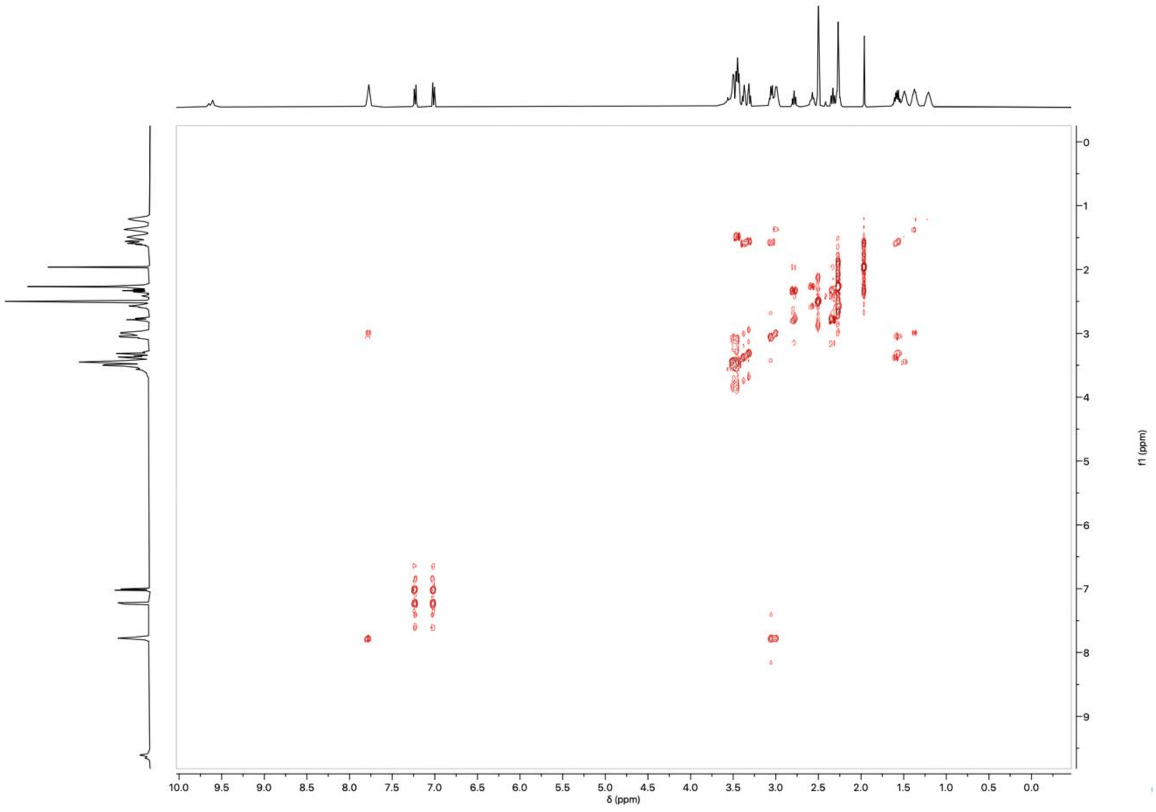

Figure S16. *2D-hetereonuclear HSQC (dmso-d^6) NMR spectrum of compound* ***1***

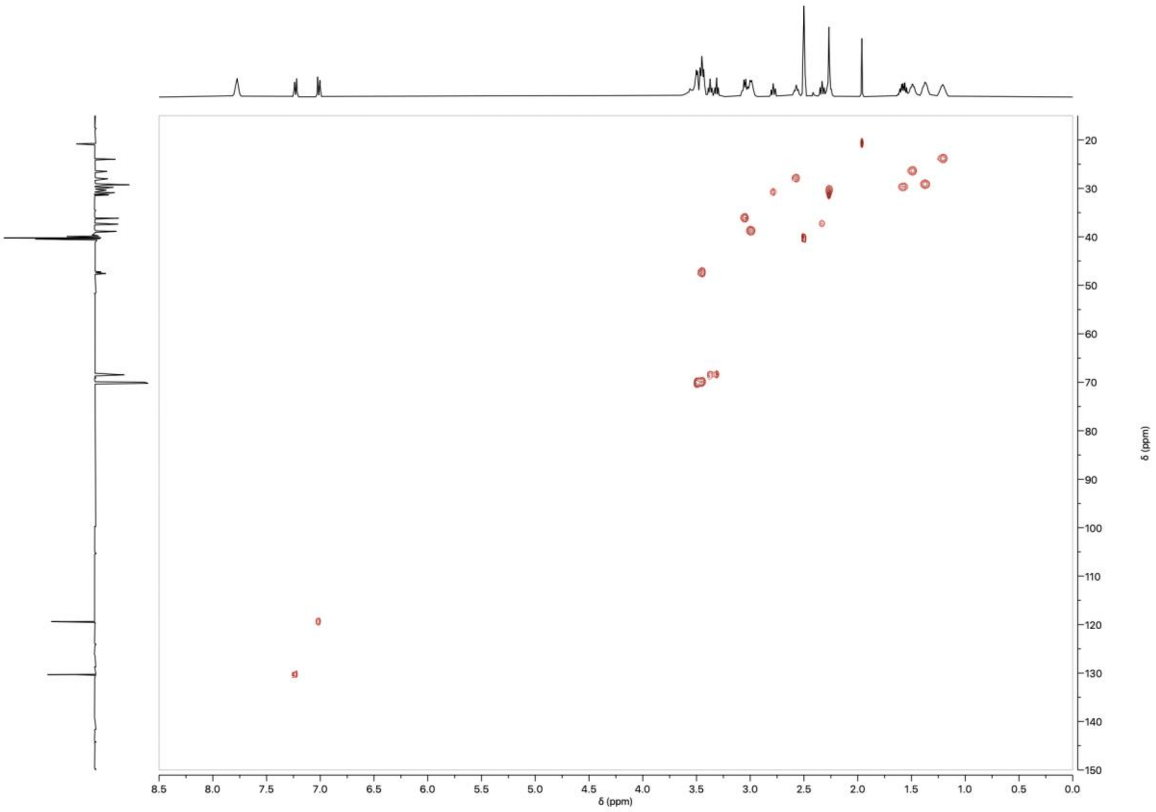

Figure S17. *HRMS spectrum of compound* ***1***

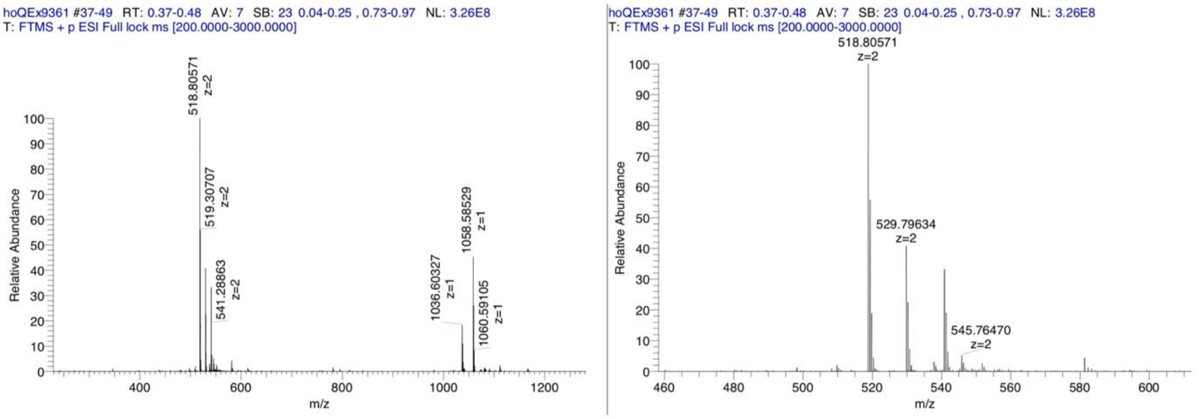

Supporting Results

^{89}Zr-Radiolabelling of DFO-PEG$_3$-Et-ArN$_3$ (1)

The [^{89}Zr]Zr-radiolabelling of DFO-PEG$_3$-Et-ArN$_3$, compound **1** to give the [^{89}Zr]Zr-radiolabelled complex [^{89}Zr]ZrDFO-PEG$_3$-Et-ArN$_3$ (^{89}Zr-**1**$^+$) was performed in triplicate. As an example, incubation of ligand **1** (20 µL; 2 mM stock solution; MW = 1036.22) with [^{89}Zr]Zr-oxalate (40 µl; 5.532 MBq) in Chelex® H_2O (40 µl) was performed in the dark for 10 mins at pH 8.0. The formation of ^{89}Zr-**1**$^+$ was confirmed by radio iTLC and radio HPLC analysis (**Figure S18**).

Figure S18. *Characterisation data for the radiochemical synthesis of [^{89}Zr]ZrDFO-PEG$_3$-Et-ArN$_3$. A) Radio-iTLC chromatograms of 'free' ^{89}Zr complexed as [^{89}Zr]Zr-DTPA (blue) and [^{89}Zr]ZrDFO-PEG$_3$-Et-ArN$_3$ (black); B) HPLC chromatograms of the ligand (compound* ***1****; blue), [^{nat}Zr]ZrDFO-PEG$_3$-Et-ArN$_3$ (green) and [^{89}Zr]ZrDFO-PEG$_3$-Et-ArN$_3$ (black).*

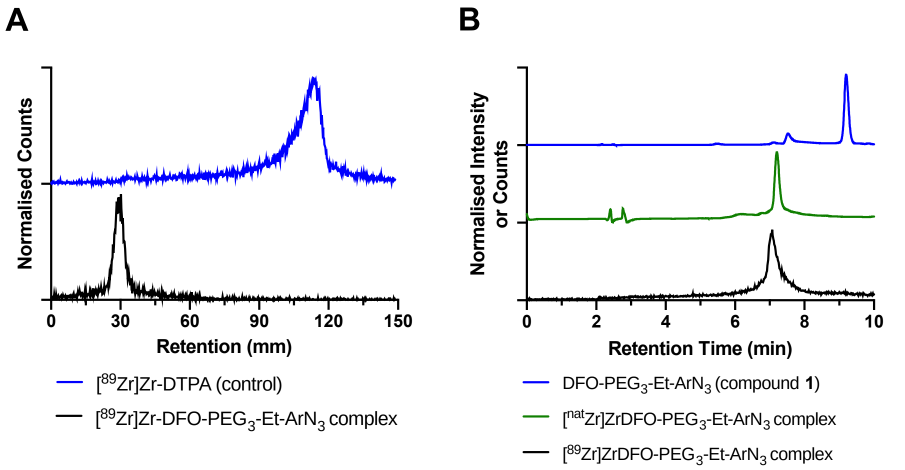

Supplement: Supplementary file 1 [file molecules-26-00764-s001.pdf]
